# Quality Control and Pre-Analysis Treatment of the Environmental Datasets Collected by an Internet Operated Deep-Sea Crawler during Its Entire 7-Year Long Deployment (2009–2016) †

**DOI:** 10.3390/s20102991

**Published:** 2020-05-25

**Authors:** Damianos Chatzievangelou, Jacopo Aguzzi, Martin Scherwath, Laurenz Thomsen

**Affiliations:** 1 Department of Physics and Earth Sciences, Jacobs University, 28759 Bremen, Germany; l.thomsen@jacobs-university.de; 2 Instituto de Ciencias del Mar (ICM-CSIC), 08003 Barcelona, Spain; jaguzzi@icm.csic.es; 3 Stazione Zoologica Anton Dohrn (SZN), 80122 Naples, Italy; 4 Ocean Networks Canada, University of Victoria, Queenswood Campus, Victoria, BC V8N 1V8, Canada; mscherwa@uvic.ca

**Keywords:** data quality, data treatment, internet operated deep-sea crawler, Barkley Canyon hydrates, Ocean Networks Canada

## Abstract

Deep-sea environmental datasets are ever-increasing in size and diversity, as technological advances lead monitoring studies towards long-term, high-frequency data acquisition protocols. This study presents examples of pre-analysis data treatment steps applied to the environmental time series collected by the Internet Operated Deep-sea Crawler “Wally” during a 7-year deployment (2009–2016) in the Barkley Canyon methane hydrates site, off Vancouver Island (BC, Canada). Pressure, temperature, electrical conductivity, flow, turbidity, and chlorophyll data were subjected to different standardizing, normalizing, and de-trending methods on a case-by-case basis, depending on the nature of the treated variable and the range and scale of the values provided by each of the different sensors. The final pressure, temperature, and electrical conductivity (transformed to practical salinity) datasets are ready for use. On the other hand, in the cases of flow, turbidity, and chlorophyll, further in-depth processing, in tandem with data describing the movement and position of the crawler, will be needed in order to filter out all possible effects of the latter. Our work evidences challenges and solutions in multiparametric data acquisition and quality control and ensures that a big step is taken so that the available environmental data meet high quality standards and facilitate the production of reliable scientific results.

## 1. Introduction

Our spatio-temporal sampling and observational capabilities are limiting our knowledge of most deep-sea environments [1,2]. Long-term time series at frequencies matching biological time-scales are essential in order to expand our understanding of highly complex physical, geochemical and biological phenomena [3,4,5]. The issue of the reliability of reference data has been brought up as imperative, in order to avoid biases at the time of parametrization and modeling of large-scale processes [6,7,8]. As datasets are getting bigger and more diverse, data collection, storage, a posteriori treatment, analysis, and visualization have to be standardized within a nationally and globally coordinated, integrated plan [9,10,11,12,13,14,15,16,17], going towards a future with automated analyses taking over from traditional, manual data treatment [18,19,20]. In this framework, communication and collaboration among scientists, engineers, and experts in the respective technological field is the only way forward in order to tackle the challenges rising from local groups working individually [21].

**Figure 1:**
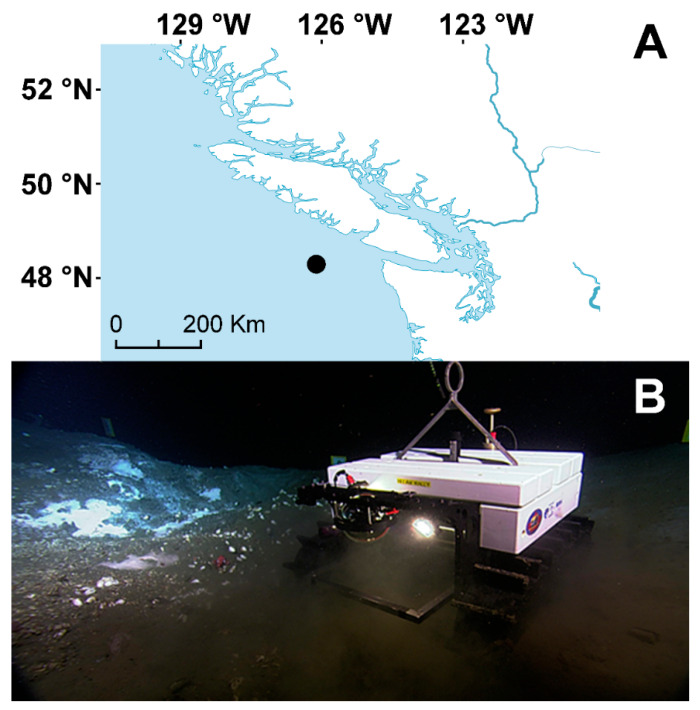
The location of the Barkley Canyon hydrates site (**A**) and the crawler (**B**).

Internet operated deep-sea crawlers represent a novel type of mobile platforms, connectable to cabled observatories, that extend the spatial coverage around the fixed node installations on the ocean floor; hence, expanding the ecological representational power of all acquired data [22,23,24]. They provide high-frequency, multi-sensor oceanographic readings, during very long-term deployments (from months to years), with a remote 24/7 communication capability. Here, expanding on the work published in [25], we present the environmental datasets obtained between late 2009 and late 2016 by the instruments mounted on the crawler “Wally”, deployed at the Barkley Canyon methane hydrates site (NE Pacific, BC, Canada; ~870 m depth, Figure 1) and connected to the Ocean Networks Canada NEPTUNE cabled observatory network (ONC; www.oceannetworks.ca), along with technical difficulties in data acquisition, quality control, and processing. All raw data are archived in near real-time, and can be accessed online on the Ocean Networks Canada database through the “Oceans 2.0” interface (https://data.oceannetworks.ca/DataSearch).

## 2. Materials and Methods

### 2.1. The Crawler and the Study Site

The crawler is a compact, mobile platform moving on caterpillars, designed for optimal transport, and handling onboard small research vessels and deployment with large 6000 m depth rated ROVs (i.e., Remotely Operated Vehicles). Power supply, communication with the remote user, and data transfer go through an umbilical cable connected to a central seafloor junction box that is connected to the Barkley Canyon node. The sensor payload included an ADM-Elektronik mini-CTD (i.e., Conductivity-Temperature-Depth), a Nortek Aquadopp Profiler, an Hs Engineers Current Meter, a Seapoint fluorometer, and a Seapoint turbidity meter. A detailed description of the crawler specifications can be found in [23].

The crawler operated at one of the gas hydrate sites of the NEPTUNE Cabled Observatory network (www.oceannetworks.ca), located on a small (1 Km^2^) plateau in Barkley Canyon (Figure 1; 48° 18′ 46′′ N, 126° 03′ 57′′ W), at approximately 870 m depth. Authorization for conducting research was provided by Transport Canada (www.tc.gc.ca/), after Fisheries and Oceans Canada (http://www.dfo-mpo.gc.ca/) assessed that the installation would not negatively impact the fish habitat.

Tides in the area are known to follow a mixed semi-diurnal pattern [26], as expected for British Columbia at the latitudes of Vancouver Island [27]. The typical range of temperature at similar depths of Barkley Canyon lies within 3.5–4.3 °C, with practical salinity reported values of 34.25–34.40 psu, while both signals are characterized by marked tidal and seasonal cycles [26,28]. Near-bottom currents rarely exceed 0.30 m/s, with the mean flow direction being towards southwest, following the general direction of the canyon [28,29]. Finally, although particle and chlorophyll signals do not present a clear seasonal periodicity and tend to follow more stochastic patterns [26], short and strong incoming chlorophyll pulses can be common from December to March [29,30], before the arrival of the more persistent, late spring and summer phytoplankton blooms.

### 2.2. Data Collection, Quality Control, and Treatment

The presented datasets contain some of the main oceanographic variables collected by the crawler sensors during the deployment period between December 2009 and December 2016. These consist of hourly averages ±SD for pressure (dbar), temperature (°C), conductivity (S/m), and practical salinity (psu), current magnitude (m/s), current direction (°), turbidity (Formazin Turbidity Units, FTU), and chlorophyll concentration (μg/l). All data values when downloaded from Oceans 2.0 are accompanied by quality flags assigned after the implementation of a series of tests, following Ocean Networks Canada’s “Quality Assurance and Quality Control (QAQC)” procedure, described more analytically online at https://www.oceannetworks.ca/data-tools/data-quality. In principle, Ocean Networks Canada adheres to the guidelines of the Quality Assurance of Real Time Oceanographic Data (QARTOD) group, whereby, after the instrument responses are parsed to archived measurements through calibration formulae, they are then automatically checked for quality in near real-time, and then also manually checked by a qualified person on a regular (mostly daily) basis. This ensures that the clean data fall within the instrument range specifications, regional, and local environmentally meaningful ranges, or are not accidentally stuck on the same value. In particular:

- Instrument Level tests (real-time) can indicate sensor failure or a loss of calibration.
- Regional Level tests (real-time) identify extreme values not associated with North East Pacific waters below 300 m depth, possibly due to sensor drift or biofouling.
- Station Level tests (real-time) further narrow the acceptable data range based on previous, adequate crawler data.
- Spike tests (delayed-mode) based on the result of Equation (1) not exceeding a variable-specific threshold
  |V_t2_ − (V_t3_ + V_t1_)/2| − |(V_t3_ − V_t1_)/2|, (1)
  with V being the value of the tested variable at three consecutive time slots t1, t2, and t3.
- Gradient tests (delayed-mode) based on the result of Equation (2) not exceeding a variable-specific threshold.
  |V_2_ − (V_3_ + V_1_)/2|, (2)
  with V being the value of the tested variable at three consecutive time slots t1, t2, and t3.
- Stuck Value tests (delayed-mode) detect non-changing scalar values within a given time period.

Before calculating any data averages, quality assurance also checks that a minimum amount of clean data is available for a meaningful standard deviation value. If the data quality control cannot assure the data to be good, the data are flagged as either “probably bad” or “bad” (with quality flags 3 or 4) dependent on the severity of the deviation. Whereas raw data contain all data with their respected quality flags, the clean data have the “probably bad” or “bad” data removed and contain data gaps instead. All original (clean) data used for the study can be downloaded online through the Oceans 2.0 interface and are also available in Supplementary Table S1.

Nevertheless, a first visual screening of the downloaded time series and their further examination revealed a set of potentially problematic issues for many observations, including:

- Absence of quality control (quality flag 0).
- Differential range and scale between distinct sensors and deployment periods for the same variable.
- Presence of underlying short- or long-term trends in values.
- Presence of non-realistic peaks and lows in values.

These issues typically stem from an absence of test criteria, e.g., through lack of documentation or experience, or potential change in actual instrument response and original calibrations, especially after instrument shipment and deployment, or data spikes within instrument ranges but with unknown expectations for the local environment, or initially unrealistic expectations, or simply sensor drifting, contamination, or fouling.

In such situations, additional manual treatment of the data was performed to make them available for use in any analysis aiming to assess the environmental conditions at the site. Firstly, the source causing the problem was identified, having in mind the particular characteristics of the study site and of the monitoring platform, as well as the expected behavior of the variable signals (e.g., by comparison to adjacent sites as provided by nearby cabled observatory platforms of the NEPTUNE network, to which the crawler is tethered). Each individual hourly observation was checked with a second-order coefficient of variation (V_2_), an alternative moment-based summary statistic that efficiently tackles many of the limitations of Pearson’s coefficient of variation (V) [31]. Subsequently, different methods of quality evaluation and treatment were used, based on the particularities of each variable and its corresponding signal and are presented below.

#### 2.2.1. Pressure

The original time series consisted of distinct deployment periods of different instruments, which translated to seven main temporal windows with visible differential scales. In particular, pressure data were obtained by the current meter (December 2009 to September 2010), by the CTD during five distinct deployments (September 2010 to July 2011, September 2011 to May 2012, June to July 2012, May 2014 to January 2015, and May to December 2016) and, finally, by the Aquadopp Profiler (July 2012 to May 2014). In addition, the data contained a considerable amount of noise. The following procedures were applied in order to obtain a smooth, correctly scaled tidal signal.

All data gaps of length 1 observation (i.e., 1 hourly missing value) were interpolated, using the mean of the adjacent observations. In continuation, the first differences of the pressure data were used to remove the majority of trends and steps. First-differenced data were modeled with the use of the R package “oce” [32], to extract the diurnal and semi-diurnal components dominating the local mixed internal tidal regime [33], as described in [34]. Then, a non-parametric, eigenvalue-based method (one-dimensional Singular Spectrum Analysis; 1D-SSA [35]) was applied to remove any underlying trends from the cumulative sum of the modeled time series. The time series were broken down to 50 periodic, trend, and random components, with the prevailing frequencies identified and used for the final signal reconstruction. This last step (i.e., decomposition and reconstruction) was performed with the R package “Rssa” [36]. Finally, the original data gaps were restored in the reconstructed time series.

#### 2.2.2. Temperature

Temperature from the first deployment window (i.e., December 2009 to September 2010, obtained by the current meter) were visibly scaled-down in comparison to the rest of the data, which originated from the CTD and the Aquadopp Profiler (for detailed information of the deployment periods see Section 2.2.1. “Pressure” above). These poorly scaled data were adjusted by adding a constant, so that the difference of the means between the two successive deployments corresponded to the difference of the means between the same temporal windows in temperature measured in an adjacent NEPTUNE site (i.e., Mid-Canyon East; 890 m depth). The exact relationship between the temperatures of the two sites during the subsequent deployment (i.e., September 2010 to July 2011) was further tested by fitting linear models in rolling 24 h-wide windows of step 1 h, in order to assess the possibility of back-calculating the bad data based on the Mid-Canyon East temperature.

#### 2.2.3. Conductivity and Salinity

The dataset originated from two sources (i.e., current meter until September 2010 and CTD from then on), with differential scaling, irregular trends, and unrealistic spikes compromising stationarity both within each particular individual deployment and universally across them all. Starting with the only stationary subset (i.e., deployment in 2014–2015), a linear model between electrical conductivity and temperature was fitted. Then, conductivity was back-calculated based on temperature for the entire 7-year span. Salinity was calculated from the new pressure, temperature, and conductivity data following the Thermodynamic Equation of Seawater-2010 (TEOS-10; [37,38]), using the R package “oce”.

#### 2.2.4. Flow

Flow data were provided by the current meter in two separate deployments (i.e., December 2009 to September 2010 and September 2011 to May 2012), with the rest of the data originating from the Aquadopp Profiler (i.e., September 2010 to July 2011 and all post-May 2012 data). Unrealistically big spikes were removed from the Aquadopp time series with the use of histograms, with outliers being defined as data belonging to the tail classes outside the first empty class on each tail. Where applicable, Cartesian coordinate (i.e., E and N) components were transformed to Euclidean vector (i.e., magnitude and direction originating from X and Y components) to facilitate comparisons between the two data formats provided by the current meter with polar plots. Magnitudes were calculated with a simple Pythagorean theorem, while the calculation of directions was conducted with the R package “circular” [39]. Finally, different deployments were compared in terms of angular dispersion around the circular mean and homogeneity, both visually and statistically (i.e., with Wallraff rank sum test of angular distance and Watson–Wheeler test for homogeneity of angles; both performed in the R package “circular”).

#### 2.2.5. Turbidity and Chlorophyll

For periods with unrealistically scaled observations, the initial electrical output (i.e., voltage) of the sensors was back-calculated and new calibration coefficients were applied to transform voltage output of the turbidity meter and the fluorometer to Formazin Turbidity Units (FTU) and μg/l, respectively. Periods with negative chlorophyll readings were centered by adding the absolute minimum value to all values of the corresponding timeframe.

## 3. Results

The complete, processed time series for all variables are available in Supplementary Table S2. From a total of 61,344 h potentially available for monitoring between December 2009 and December 2016, 14,949 h (24.37%) corresponded to universal data gaps (i.e., missing values across all variables), meaning that for 75.63% of the monitoring period there was at least one variable returning a useable value. In total, out of 490,752 potentially available time-slots (i.e., 61,344 h × 8 variables), there were 203,730 missing values (41.51%). Details for each variable are provided in the corresponding subsection below.

### 3.1. Pressure

**Figure 2:**
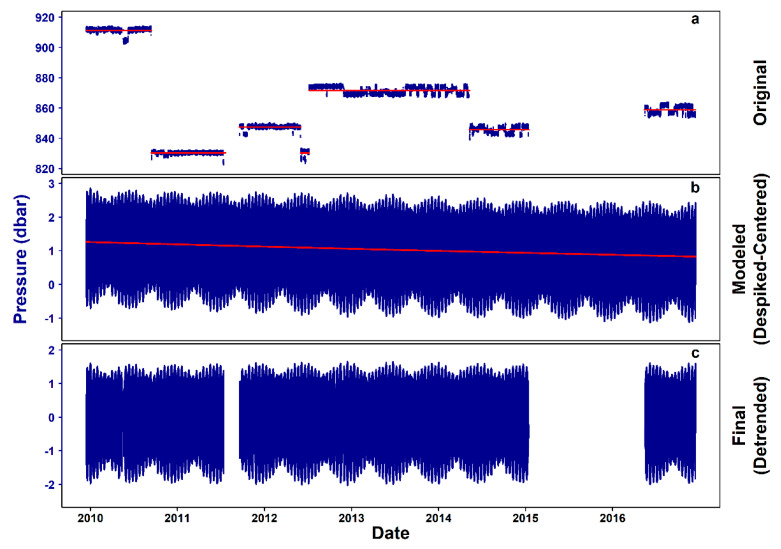
Steps of pressure data processing. (**a**) Original 7-year time series (i.e., December 2009–December 2016), with red lines indicating the pressure mean in each temporal window, (**b**) cumulative sum of the model-predicted differences, with data gaps filled to facilitate the Singular Spectrum Analysis (SSA) and the red line indicating the underlying linear trend and finally, (**c**) clean time series, with the original data gaps restored.

The original hourly pressure observations had a coefficient of V_2_ ranging from 4.14 × 10^−5^ to 3.03 × 10^−3^ (“very small” as per [31]). The original time series, containing deployment periods with differential scales as well as noise, are presented in Figure 2a.

**Figure A1:**
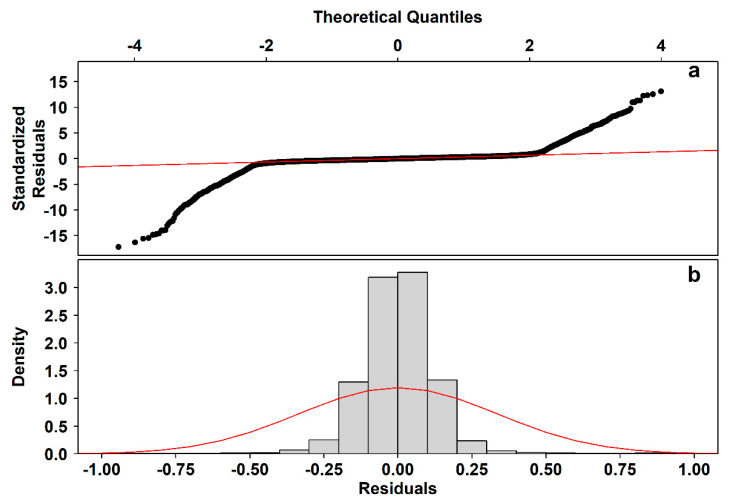
Graphical analysis of the tidal model residuals. (**a**) Quantile-quantile plot of standardized residuals vs. theoretical quantiles, with the red normality line, (**b**) histogram of the residuals with a red normal distribution curve. Both plates are scaled for visual purposes, to exclude extreme outliers that would make the figure hard to read.

Table 1 presents the diurnal and semi-diurnal tidal components extracted from the modeled differenced data. The residuals of the model are further analyzed in the Appendix A (Figure A1; Appendix A.1. Tidal Model Residual Analysis), while the complete model output is available in detail in Supplementary Table S3.

The cumulative sum of the model outcome (moving up from the first differences after the noise deduction) still contained a slight linear decreasing trend (Figure 2b), which was removed by applying the 1D-SSA, resulting in the final, stationary signal (Figure 2c). Moreover, 15,053 values (24.54%) were missing from the final pressure time series.

### 3.2. Temperature

**Figure 3:**
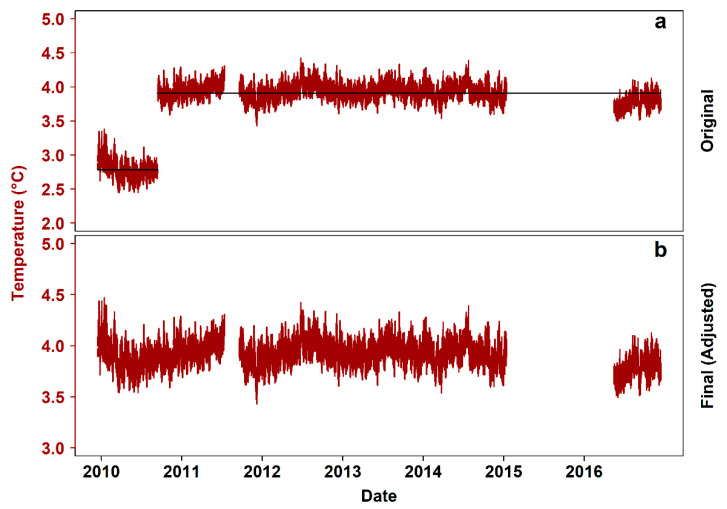
Steps of temperature data processing. (**a**) Original 7-year time series (i.e., December 2009−December 2016), with black lines indicating the mean in each temporal window, (**b**) clean time series after centering.

**Figure A2:**
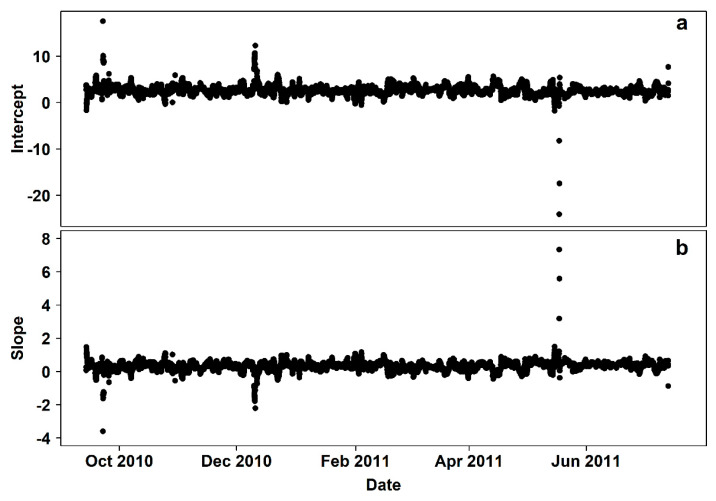
Graphical analysis of the hydrates–Mid-Canyon East temperature model coefficients. (**a**) Time series of the intercept, (**b**) time series of the slope. Each point corresponds to a linear model fitted to a 24 h-wide window.

V_2_ for hourly temperature observations ranged from 0 to 5.35 × 10^−2^ (“very small”). The temperature means between two successive deployments (i.e., switch in September 2010) differed by 1.16 °C (Figure 3a). Figure 3b presents the final, adjusted temperature time series, after the unrealistically low pre-September 2010 data were moved up so that the aforementioned difference was reduced to ~0.07 °C (i.e., the corresponding temperature difference between the same temporal windows in an adjacent site), with a total of 15,053 values missing (24.54%). The linear relationship between data from hydrates site and the nearby Mid-Canyon East site varied in time (Figure A2; Appendix A.2. Hydrates – Mid-Canyon East Temperature Comparison), leading to the exclusion of using Mid-Canyon East temperatures to back-calculate the bad, pre-September 2010 data.

### 3.3. Conductivity and Salinity

**Figure 4:**
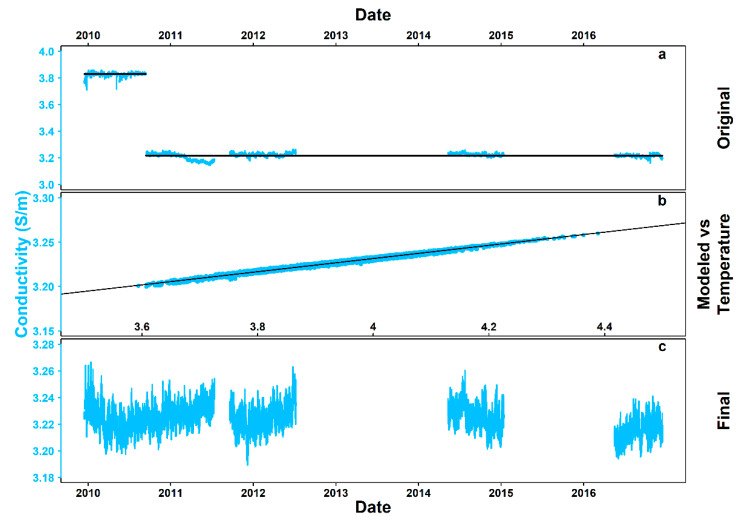
Steps of conductivity data processing. (**a**) original 7-year time series (i.e., December 2009–December 2016), with black lines indicating the mean in each temporal window, (**b**) linear relationship between conductivity and temperature and finally, (**c**) model-predicted time series.

Hourly conductivity time series had a V_2_ from 2.36 × 10^−5^ to 2.04 × 10^−2^ (“very small”) and contained different means, irregular trends, and spikes (Figure 4a).

**Figure A3:**
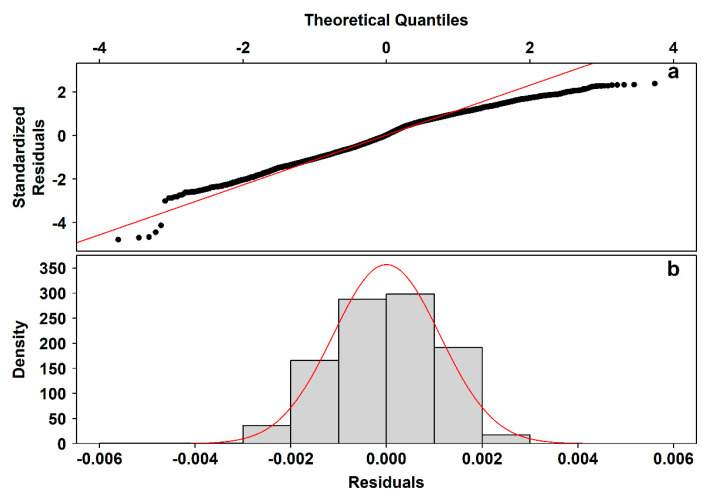
Graphical analysis of the conductivity – temperature model residuals. (**a**) Quantile-quantile plot of standardized residuals vs. theoretical quantiles, with the red normality line, (**b**) histogram of the residuals with a red normal distribution curve.

Figure 4b presents the linear relationship between conductivity and temperature for the stationary subset May 2014 to January 2015, described in this case by Equation (3):

EC = 0.07t + 2.93,(3)

with adjusted R^2^ = 0.98, *p* < 2.2 × 10^−16^, F statistic = 3.2 × 10^5^ (5392 *df*). EC stands for electrical conductivity and t for temperature. The residuals of the linear model are further analyzed in the Appendix A (Figure A3; Appendix A.3. Conductivity – Temperature Model Residual Analysis).

The final, back-calculated conductivity time series, with 30,533 values missing (49.77%), are presented in Figure 4c.

### 3.4. Flow

**Figure 5:**
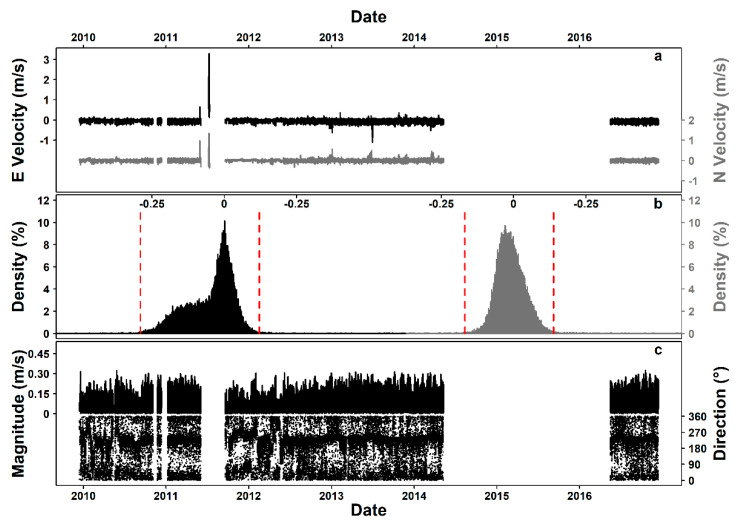
Steps of flow data processing. (**a**) Original 7-year time series (i.e., December 2009–December 2016) of E (East) and N (North) flow components (i.e., black for E and gray for N), (**b**) histograms for each component for the despiking of the Aquadopp data and finally, (**c**) complete time series of flow magnitude and direction.

The original E and N (i.e., East and North) components were characterized by V_2_ from “very small” to “very large” (i.e., from 7.42 × 10^−2^ to 1 and from 9.13 × 10^−2^ to 1, respectively) and presented unrealistically big spikes affecting the scale and range of the time series (Figure 5a). Figure 5b presents the cut-off points for outliers on each tail of the respective histograms.

**Figure A4:**
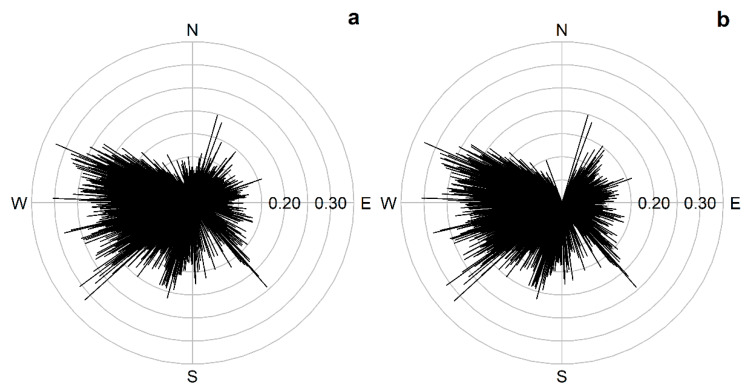
Polar plots of current meter flow data. (**a**) Magnitude and direction derived from E–N components, (**b**) magnitude and direction derived from X–Y components. The labels in the grid indicate magnitudes in m/s.

The polar plots comparing E–N component to X–Y component data are provided in the Appendix A (Figure A4; Appendix A.4. Current Meter Flow Component Comparison), with a ~36° gap in the north part of the spectrum (340°–16°) notable in the latter.

The complete time series had 23,135 missing values (37.71%) per variable, and presented visual (Figure 5c) and statistical (Wallraff and Watson–Wheeler tests; Table 2) differences along time in both angular dispersion around the circular mean and homogeneity.

### 3.5. Turbidity and Chlorophyll

**Figure 6:**
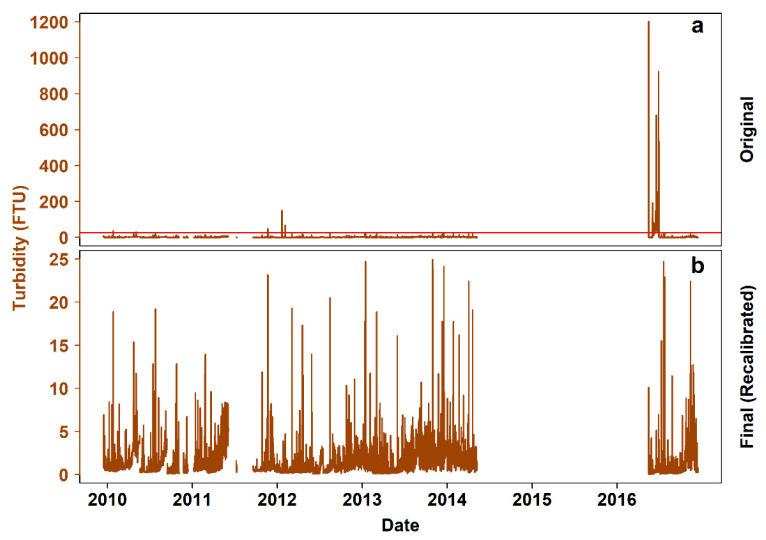
Steps of turbidity data processing. (**a**) Original 7-year time series (i.e., December 2009–December 2016), with the red line indicating the sensor’s theoretical maximum reading (i.e., 25 Formazin Turbidity Units (FTU), based on the selected sensitivity and range settings applied before deployment), (**b**) clean time series, after back-calculating the sensor’s electrical output and applying the correct calibration coefficients.

V_2_ for turbidity also ranged from “very small” to “very large” (i.e., from 3.15 × 10^−6^ to 1). Figure 6a presents the original time series, with unrealistically high values in early 2012 and mid-2016. In Figure 6b, these values have been either corrected (i.e., recalculated with the use of correct calibration coefficients) or eliminated from the final time series, with a total of 22,890 values missing (37.31%).

**Figure 7:**
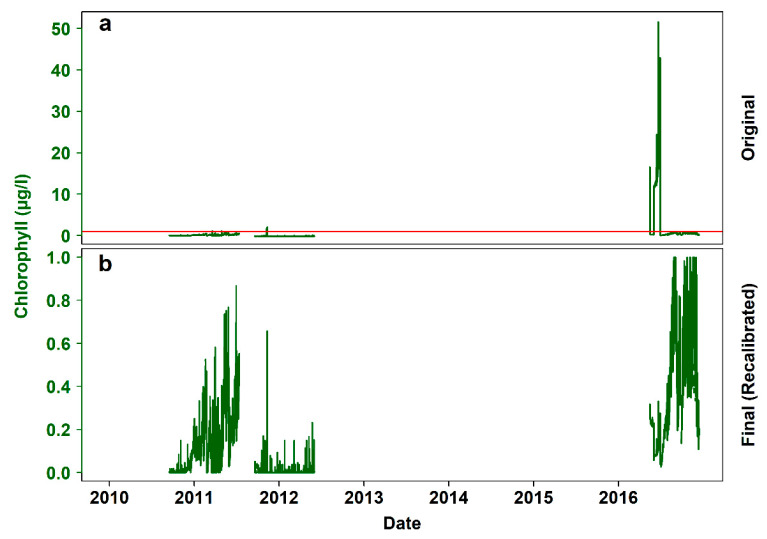
Steps of chlorophyll data processing. (**a**) Original 7-year time series (i.e., December 2009 – December 2016), with the red line indicating the sensor’s theoretical maximum reading (i.e., 1 μg/L, based on the selected sensitivity and range settings applied before deployment), (**b**) processed time series, after back-calculating the sensor’s electrical output and applying the correct calibration coefficients.

In regards to chlorophyll, the behavior of V_2_ was similar (i.e., from “very small” to “very large”; 0 to 1). The original time series (Figure 7a) also contained unrealistically high values, as well as negative values. In Figure 7b, the wrongly scaled values have been corrected with new coefficients or eliminated from the final time series, the subsets with negative minimum readings (September 2010 to April 2011 and September 2011 to May 2012) have been centered, but there is still evidence of underlying local trends. In total, 43,398 values were missing (70.75%).

## 4. Discussion

### 4.1. General Remarks

The quality of the 7-year time series for pressure, temperature, electrical conductivity, current flow, turbidity, and chlorophyll, as collected by an Internet Operated Deep-sea Crawler at the Barkley Canyon hydrates site between 2009 and 2016, was assessed, and necessary processing steps were taken to tackle any underlying issues.

Starting with data availability, the universal data gaps corresponded to either periods when the crawler was not deployed (most notably July to September 2011 and January 2015 to May 2016), periods of adjustment (e.g., first hours after deployment or in situ maintenance by ROV during regular Ocean Networks Canada maintenance cruises), or general power outages of the observatory (i.e., either for planned maintenance or due to unexpected events). On the other hand, differences in gaps among individual variables are related to the data quality of a particular variable for a given period (i.e., data discarded after being labeled as “probably bad” or “bad”) or simply to periods when the corresponding instrument was not deployed. For instance, no conductivity data were available between July 2012 and May 2014 due to the absence of a CTD, with temperature and pressure available through the Aquadopp Profiler, which did not report conductivity). On a similar note, there were no flow data from May 2014 to January 2015 (i.e., no profiler or current meter) and no chlorophyll data from June 2012 to January 2015.

In regards to data assessment, extensive natural variability ranging from short-term fluctuations to pronounced inter-annual differences can be expected, and is acceptable for the oceanographic variables assessed here (e.g., a period of high detrital input or an unusually cold year). In that sense, the loose term “non-stationarity” was used to describe only the lack of stationarity due to external factors (i.e., related to the performance of the instruments and the operations of the crawler as a mobile monitoring platform).

The variables that presented potentially problematic levels of the second-order coefficient of variation V_2_ (i.e., hourly averages from highly varying, high-frequency observations) were flow, turbidity, and chlorophyll. Although with such results the quality of the hourly averaged data may appear compromised, this can be expected for highly dynamic variables such as currents, which in turn strongly affect particle and phytodetritus concentrations. Flow characteristics can vary down to temporal scales of minutes [40], in contrast to more stable seawater properties (e.g., temperature and conductivity). The hourly averages are an indication of the general flow during the corresponding temporal window. However, short-term opposite flows throughout an hour may not be fully reflected, as they could potentially be cancelled out while averaging, resulting in high variation. Pressure, finally, could change abruptly during an hour, due to the movement of the crawler across a depth gradient, with the depth difference within the operational range of the crawler reaching up to 10 m. Nevertheless, such differences are not deemed significant at these depths, so the standard deviations were not affected to such a degree in order to raise the V_2_ values to compromising levels.

### 4.2. Remarks on Individual Environmental Variables

The visually apparent presence of differential scale and noise in pressure signal throughout the time series was a product of the use of three different data sources (i.e., Aquadopp Profiler, current meter, and CTD) and of the displacement of the crawler through parts of the Barkley Canyon hydrates seafloor with steep morphological features and different depth, respectively. These effects had to be removed for the tidal signals to be usable. After a conservative gap filling, we used first-differenced data for modeling, based on the principle that the first differences of a sine wave maintain the frequencies of the original. The use of one-dimensional Singular Spectrum Analysis (1D-SSA) successfully removed the final underlying trend while it maintained the periodic and random elements of the signal, as the same frequencies as in the model were detected. The amplitude of the final signal was similar to the pressure signal of fixed sensors deployed at different instrument platforms in Barkley Canyon [16].

Temperature non-stationarity was a result of different data sources (i.e., Aquadopp Profiler, current meter, and CTD), with the current meter data being unrealistically scaled-down for these depths. After adjustment, the signal compares well to the temperature from other instruments deployed at the hydrates and from adjacent Barkley Canyon instrument platforms [28]. No scaling was necessary, as the range of the time series did not change in time between the deployment periods in question. This type of approach was adopted instead of other, potentially more robust in a pure statistical sense (e.g., modeling and backcasting the temperature data based on the crawler CTD time series, or modeling the crawler CTD time series against temperature from other sites and back-calculate temperature for that period when the current meter was deployed), due to the particularities of this specific case study. The often spatiotemporally auto-correlated nature of temperature data can lead to high uncertainty of backcasted values [41]. That uncertainty would accompany any further analysis, even though the actual temperature values could exist within the prediction intervals of an ARIMA (i.e., Auto Regressive Integrated Moving Average) family model. The use of different instruments and spatial heterogeneity were the main reasons for rejecting the option to back-calculate the data based on temperature of another site. Temperatures of sites located at similar depths of Barkley Canyon, such as the hydrates site (where the crawler was deployed) and the adjacent Mid-Canyon East site, are expected to be roughly within the same ranges and follow the same seasonal patterns, as can be observed by comparing the present study with long-term data presented in [28]. Nevertheless, that does not account for sharp local maxima/minima due to the different geomorphological settings and consequent hydrographic scenarios of each site [42]. This was corroborated by the visible time-dependency of the relationship between the temperature data of the two sites from September 2010 to July 2011. With both raw and processed datasets provided here along with the described processing methodology, any future work using temperature data from the crawler can further treat the data in order to fit its specific needs (e.g., modeling and prediction of the oceanographic state of a canyon [43] vs. assessment of the effect of high-frequency fluctuations of an oceanographic variable on the faunal community [44]).

In the case of conductivity, the time series contained different means per deployment and instrument (i.e., current meter and CTD), irregular trends, and spikes. Only data from the first deployment (current meter) were unrealistically high, indicating an error in the configuration of the instrument, when interpreted in combination with the downscaled temperature data from the same source. Except for the deployment from May 2014 to January 2015, in other subsets of the time series there were visible drifts pointing towards sensor failure (e.g., possible fouling by accumulation of salt around the sensor). The use of a linear model was selected to completely recalculate conductivity from temperature (i.e., a variable without reasons for rejection of the data), as the relationship between the two is expected to be linear in the temperature range for environmental monitoring (i.e., 0–30 °C) [45]. Indeed, the fit of the model was near perfect, allowing conductivity to be back-calculated based on temperature for the entire 7-year span. The linear fit and the resulting time series compared well to the respective data from adjacent platforms [28], adding further value to the method.

Current flow data, in the form of two Cartesian velocity components, E and N, originated from two different instruments (i.e., current meter and Aquadopp Profiler), with the profiler data presenting unrealistically big spikes, affecting the scale and range of the time series. The current meter also provided data in Euclidean vector format (i.e., magnitude and direction originating from X and Y components). E and N components were transformed to vector format for comparison (i.e., polar plots), based on which the X–Y originated data were discarded. Differences in terms of angular dispersion around the circular mean and homogeneity among all deployments made any posterior adjustments of the data, apart from despiking, impossible within the framework of the current study. For that purpose, an inspection of the positional attributes of the sensors (i.e., pitch and roll) and magnetometer data could be a future step.

Calibration issues with the turbidity and chlorophyll sensors lead to erratically scaled data for a 30-day period (i.e., June 2016). After back-calculating the original electrical outputs of the sensors, the values were recalculated and compared well to data from adjacent platforms. Nevertheless, there were still apparent local trends in both time series. These could either be real particle or chlorophyll incoming pulses, or artificial (i.e., attributed either to biofouling, in which case the data will have to be discarded, or to the operational protocol of the crawler during its mission in the corresponding period of time). For example, the signals of these two optical sensors can be expected to be affected by the crawler’s movement and the resulting resuspended sediment [29,46]. To filter such effects, the signals have to be assessed in parallel with data on the movement and operations of the crawler.

### 4.3. Automated and Manual Data Quality Control and Validation

The differences in the issues compromising the quality of each time series, along with the particularities of each variable, pointed towards a case-by-case approach in this study. In this context, the applied treatment method was decided based on the combinations of characteristics such as:

- Are subsets of the time series wrongly scaled?
- Are there implausible gradients in the time series?
- Do the time series contain implausible spikes?
- Are the time series compromised by unnatural noise?
- Is the variable characterized by marked periodicities or other patterns?
- Can the variable time series be modeled?
- Are the values of the variable positive by definition or do they range in the entire ℝ (i.e., real numbers) field?

**Figure 8:**
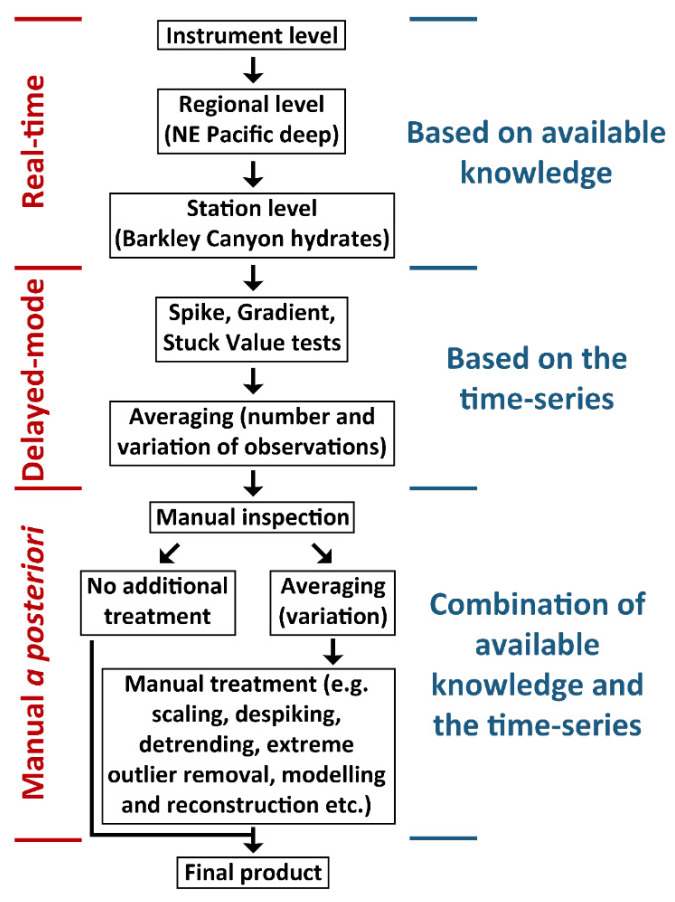
Flowchart of the quality control and treatment procedures. Red and blue labels indicate the timing of the process and the basis of the criteria used, respectively. The first five steps (automated) are performed either natively in the instrument, before the data are uploaded on the Oceans 2.0 database or before data are downloaded by the user.

The increasing volume of incoming data needed in order to achieve the principal goals in marine environmental monitoring and management, as they were recently identified in reports by intergovernmental entities (e.g., the Intergovernmental Science-Policy Platform on Biodiversity and Ecosystem Services, IPBES and the Intergovernmental Panel on Climate Change, ICCP [47,48]), demands more automation and standardization in data quality control and treatment. In Figure 8, a schematic description of the hybrid, semi-automated approach followed in this study is provided, based on the adherence of Ocean Networks Canada to the QARTOD guidelines for data management. Manual evaluation and treatment complemented or substituted the automated (real-time and delayed-mode) QAQC steps described in Section 2.2. “Data Collection, Quality Control and Treatment” because automated quality control was incomplete or not performed (quality flag 0) but regular manual inspection by a data expert is recommended for final validation, irrespectively. Real-time, automated tests are based on previous knowledge and can be applied to any individual value without a need for either its adjacent values or the corresponding subset of the time series. On the other hand, delayed-mode automated tests consist of sliding window techniques without taking into account the context of the actual values, and are therefore based entirely on the statistical behavior of the time series. For the manual a posteriori treatment, which was the focus of this study, a combination of both approaches had to be taken into account in order to tackle any underlying issues with the time series.

### 4.4. Future Steps and Perspectives

The present study provided the additional, manual steps required to assure the better usability of the environmental datasets collected by the crawler between 2009 and 2016. The natural evolution of this work would include the integration of operational and positional crawler data for the better interpretation of the environmental data, and a complete evaluation of the performance of each instrument and sensor (e.g., quantitative and qualitative assessment, comparisons with the performance of any corresponding infrastructure of other Barkley Canyon sites), as well as the development of semi-automated routines for the application of such analyses.

## 5. Conclusions

In the developing era of integrated strategies in deep-sea monitoring, assurances of data quality and comparability in space (e.g., different sites) and time (e.g., different deployments) are crucial, and require adequate documentation of all the procedures preceding the use, sharing, and publication of datasets, including data collection, quality control, and treatment. Even though some specific steps can vary among variables and sites, following protocols as, in this case, the Ocean Network Canada’s “Quality Assurance and Quality Control (QAQC)”, based on internationally accepted guidelines, such as the ones provided by the Quality Assurance of Real Time Oceanographic Data (QARTOD) group, is of paramount importance. Further integration of such steps will be highly aided by the increasing development of Artificial Intelligence and automation, although manual inspection of data on a regular basis should not be discarded.

## Figures and Tables

**Table 1:** Tidal constituents identified by modeling of the differenced pressure data. Constituent characterization based on [34].

Type	Constituent	Period
lunar diurnal	O1	25.82
solar diurnal	P1	24.07
lunar diurnal	K1	23.93
smaller lunarelliptic diurnal	J1	23.10
lunar ellipticalsemi-diurnal second-order	2N2	12.91
larger lunar evectional	NU2	12.63
principal lunarsemi-diurnal	M2	12.42
principal solarsemi-diurnal	S2	12.00

**Table 2:** Statistical comparison of flow data between deployments of different instruments.

Test	Statistic	*p* Value
Wallraff	373.2 (4 *df*)	< 2.2 × 10^−16^
Watson-Wheeler	13.68 (2 *df*)	1.07 × 10^−3^

## Supplementary Materials

The following are available online at https://www.mdpi.com/1424-8220/20/10/2991/s1.

## Appendix A

### Appendix A.1. Tidal Model Residual Analysis

The analysis of the tidal model residuals revealed heavy tails in the quantile–quantile plot (i.e., standardized residuals vs. theoretical quantiles; Figure A1a) and a narrow density histogram (Figure A1b), although the behavior of the middle range approximates normality. Such behavior would indicate the existence of some outliers in an otherwise robust fit, an assumption further strengthened by the independence of the residuals from the fitted values.

### Appendix A.2. Hydrates—Mid-Canyon East Temperature Comparison

The time series of the rolling coefficients showed a time-dependency in the relationship between temperature data from the two sites, meaning that there is no equivalence in short temporal scales (i.e., hours). Based on this, back-calculating pre-September 2010 temperature based on the Mid-Canyon East data was not considered adequate.

### Appendix A.3. Conductivity—Temperature Model Residual Analysis

The histogram of the residuals generally followed the normal distribution curve (Figure A3b), however the left tail in the quantile-quantile plot (Figure A3a) revealed the presence of a few outliers, which should not affect the almost perfect fit of the model, keeping in mind the size of the dataset.

### Appendix A.4. Current Meter Flow Component Comparison

Current meter flow data originating from different components (i.e., E–N and X–Y) were compared with the use of polar plots (Figure A4). Even though the general behavior of the data was similar between the two sources, a gap is notable in North range of the X–Y data (Figure A4b), excluding them from further analysis.
